# Ventricular Tachycardia in Patients With Takotsubo Cardiomyopathy: Prevalence, Predictors, and Associated In-Hospital Adverse Events

**DOI:** 10.7759/cureus.57724

**Published:** 2024-04-06

**Authors:** Omar Elkattawy, Mabad Shacker, Sedra Alabed, Daniel Elias, Sherif Elkattawy, Omar Mohamed, Charlene Mansour, Casey Hamlet, Salma Emara, Fayez Shamoon

**Affiliations:** 1 Department of Internal Medicine, Rutgers University New Jersey Medical School, Newark, USA; 2 Department of Cardiology, St. Joseph's University Medical Center, Paterson, USA; 3 Department of Medicine, Saint Barnabas Medical Center, Livingston, USA; 4 Department of Internal Medicine, Rowan University School of Osteopathic Medicine, Stratford, USA

**Keywords:** heart failure and medical education, ventricular arrhythmia, preventative cardiology, takotsubo cardioyopathy, ventricular tachycardia (vt)

## Abstract

Introduction

The purpose of this study was to determine the prevalence of ventricular tachycardia (VT) among patients admitted with takotsubo cardiomyopathy (TCM) as well as to analyze the predictors of VT and the predictors of mortality among patients admitted with TCM.

Methods

Data were obtained from the National Inpatient Sample (NIS) database from January 2016 to December 2019. Patients with a primary diagnosis of TCM were selected using ICD-10 code I51.81. Subsequently, the study population was divided into patients who developed VT vs. patients who did not develop this complication. We then used multivariate logistic regression to assess the predictors of VT in our patient cohort as well as the predictors of mortality among patients admitted with TCM.

Results

Of 40114 patients with TCM, 1923 developed VT (4.8%) during their hospital stay. Predictors of VT include atrial fibrillation (AF) (adjusted odds ratio (aOR): 1.592; 95% confidence interval (CI): 0.00-1.424; p=0.001), congestive heart failure (aOR: 1.451; 95% CI: 1.307-1.610; p=0.001), coagulopathy (aOR: 1.436; 95% CI: 1.150-1.793; p=0.001), and patients who self-identify in the race category as Other (aOR: 1.427; 95% CI: 1.086-1.875; p=0.011). Female sex was found to be protective against VT (aOR: 0.587; 95% CI: 0.526-0.656; p=0.001). Predictors of mortality among patients admitted with TCM include, among other factors, age (aOR: 1.014; 95% CI: 1.011-1.018; p=0.001), Asian or Pacific Islander race (aOR: 1.533; 95% CI: 1.197-1.964; p=0.001), Black race (aOR: 1.242; 95% CI: 1.062-1.452; p=0.007), VT (aOR: 1.754; 95% CI: 1.505-2.045; p=0.001), and AF (aOR: 1.441; 95% CI: 1.301-1.597; p=0.001). Some comorbidities that were protective against mortality in TCM include tobacco use disorder (aOR: 0.558; 95% CI: 0.255-0.925; p=0.028) and obstructive sleep apnea (aOR: 0.803; 95% CI: 0.651-0.990; p=0.028). The female sex was found to be protective against mortality (aOR: 0.532; 95% CI: 0.480-0.590; p=0.001).

Conclusion

In a large cohort of women admitted with TCM, we found the prevalence of VT to be 4.8%. Predictors of VT included conditions such as AF and congestive heart failure. The female sex was found to be protective against VT and protective against mortality among patients admitted with TCM.

## Introduction

Takotsubo cardiomyopathy (TCM) is a form of nonischemic cardiomyopathy that is characterized by acute and transient left or right ventricular dysfunction [1]. Although the mechanisms of pathophysiology are still unclear, there has been evidence that suggests TCM commonly occurs following severe emotionally stressful events, with clinical symptoms and findings that are similar to those during an acute myocardial infarction [2]. A distinguishing factor between TCM and myocardial infarction is the absence of coagulative necrosis and the minimal release of myocardial enzymes [2]. While the prognosis can be favorable in many cases, serious complications such as life-threatening arrhythmias have been reported to occur in some hospitalized TCM patients [3]. QT interval prolongation or T-wave abnormalities are hypothesized to be involved in the development of these fatal arrhythmias, but further studies are still needed to determine the mechanism of action [4]. Some of these arrhythmias include ventricular tachycardia (VT), ventricular fibrillation (VF), and atrial fibrillation (AF).

VT is a life-threatening arrhythmia and one of the most common causes of sudden cardiac death in the United States [5]. The clinical presentation of VT can include hypotension, chest pain, and palpitations that result from a low cardiac output because of delayed after-depolarization that results in decreased preload [6]. In the presence of a prolonged QT interval and left ventricular systolic dysfunction, VT can result in hemodynamic collapse [6]. Studies have emerged describing ventricular arrhythmias to be one of the most common arrhythmias in TCM patients that can worsen long-term prognosis [7]. In this study, we aimed to examine the prevalence, predictors, and in-hospital adverse events associated with VT in TCM patients.

## Materials and methods

Data acquisition 

This is a retrospective database study of the National Inpatient Sample (NIS) database. The NIS is part of the Healthcare Cost and Utilization Project (HCUP) set forth by the Agency for Healthcare Research and Quality of the United States Department of Health and Human Services. It utilizes the International Classification of Diseases, 10th Revision, Clinical Modification (ICD-10-CM) codes for diagnosis and procedures. The data set was utilized to examine data of patients admitted from 2016 to 2019. Encounters with a primary diagnosis of TCM were selected using ICD-10 code I51.81. This cohort of patients was further divided into patients who developed VT vs. patients without VT. The diagnosis of VT was filtered based on the ICD-10 code I47.2. Adult patients ≥18 years old were included. We abstracted data from 40,138 charts, excluded 24, and were left with 40,114 charts for analysis. An IRB approval was not required as NIS provides de-identified information on patients.

Outcomes and variables 

Patient baseline characteristics such as age, sex, race, and insurance status were extracted. Comorbidities, hospital complications, mortality rates, disposition status, length of stay, and total charges were also analyzed.

The primary aim of the study was to assess the characteristics and conditions that were predictors of VT in patients admitted with TCM. We also assessed whether or not there was a difference in outcomes (mortality, in-hospital complications, length of stay, total charges) between the cohort of patients with TCM and VT and patients with TCM who did not develop VT. Lastly, we analyzed the predictors of mortality among all patients admitted with TCM. 

Statistical analysis 

Categorical values were analyzed via Pearson's chi-square analysis and continuous variables were analyzed via independent Student’s t-test. Logistic regression was performed to generate odds ratios (ORs) with 95% confidence intervals (CIs) to assess predictors of VT in women with TCM. We also used logistic regression to assess the predictors of mortality among all patients admitted with TCM. A p-value of <0.05 was considered statistically significant. All analyses were completed using IBM SPSS Statistics for Windows, Version 29, (Released 2023; IBM Corp., Armonk, New York, United States).

## Results

Of 40114 patients with TCM, 1923 (4.8%) developed VT during their hospital stay. A statistical analysis of baseline characteristics is summarized in Table 1. Age is significantly associated with VT in TCM, with individuals in the VT group being younger on average than those in the non-VT group (65.8±14.7 years vs. 67.2±14.1 years; p=0.001). On an analysis of discharge disposition, patients with VT had fewer routine discharges (719 (37.4%) vs. 20522 (53.8%); p=0.001). The female sex was the majority in both groups, but a higher percentage of men was found in the VT group compared to the non-VT group (535 (27.8%) vs. 6281 (16.4%); p=0.001). When stratified according to race, the analysis did not reveal a significant difference in the prevalence of VT among different races (p=0.078). Lastly, there was an increased prevalence of patients with Medicare in the non-VT group compared to the VT cohort (24330 (63.8%) vs. 1146 (59.6%); p=0.001). 

**Table 1:** Baseline characteristics of the study population of TCM stratified according to VT status The data has been represented as n and percentage, or mean ± SD. p-values are significant at <0.05. TCM: takotsubo cardiomyopathy; VT: ventricular tachycardia; SD: standard deviation

	Patients without VT (n=38,191)		Patients with VT (n=1,923)		p-value
Age in years at admission	67.2±14.1		65.8±14.7		0.001
Disposition of patient					0.001
Routine	20522	(53.8)	719	(37.4)	
Transfer to short-term hospitals	1044	(2.7)	74	(3.8)	
Transfer other: includes skilled nursing facility (SNF), intermediate care facility (ICF), and other types of facility	8004	(21.0)	530	(27.5)	
Home health care (HHC)	6058	(15.9)	320	(16.6)	
Against medical advice (AMA)	325	(0.9)	9	(0.5)	
Died in hospital	2227	(5.8)	272	(14.1)	
Indicator of sex					0.001
Male	6281	(16.4)	535	(27.8)	
Female	31910	(83.6)	1388	(72.2)	
Primary expected payer					0.001
Medicare	24330	(63.8)	1146	(59.6)	
Medicaid	3965	(10.4)	218	(11.3)	
Private insurance	8047	(21.1)	451	(23.4)	
Self-pay	981	(2.6)	78	(4.1)	
No charge	70	(0.2)	3	(0.2)	
Other	760	(2.0)	28	(1.5)	
Race					0.078
White	29857	(80.7)	1458	(78.7)	
Black	2979	(8.1)	160	(8.6)	
Hispanic	2323	(6.3)	122	(6.6)	
Asian or Pacific Islander	748	(2.0)	43	(2.3)	
Native American	242	(0.7)	10	(0.5)	
Other	829	(2.2)	59	(3.2)	

Univariate analysis results showing the associations between several comorbidities and VT in TCM patients are depicted in Table 2. Univariate analysis shows a higher burden of comorbidities in the VT group including AF (566 (29.4%) vs. 7703 (20.2%); p=0.001), coagulopathy (106 (5.5%) vs. 1098 (2.9%); p=0.001), cerebrovascular disease (42 (2.2%) vs. 549 (1.4%); p=0.008), alcohol use disorder (156 (8.1%) vs. 2176 (5.7%); p=0.001), liver disease (77 (4.0%) vs. 893 (2.3%); p=0.001), peripheral vascular disease (PVD) (62 (3.2%) vs. 893 (2.3%); p=0.013), pulmonary hypertension (173 (9.0%) vs. 2540 (6.6%); p=0.001), obstructive sleep apnea (OSA) (132 (6.9%) vs. 2156 (5.6%); p=0.025), and end-stage renal disease (ESRD) (56 (2.9%) vs. 762 (2.0%); p=0.006). The non-VT cohort had a higher prevalence of chronic obstructive pulmonary disease (COPD) (12869 (33.7%) vs. 577 (30.0%); p=0.001), hypertension (13998 (36.6%) vs. 512 (26.6%); p=0.001), and hypothyroidism (6828 (17.9%) vs. 266 (13.8%); p=0.001).

**Table 2:** Prevalence of comorbidities in the study population of TCM patients with and without VT The data has been represented as n (%). p-values are significant at <0.05. VT: ventricular tachycardia; TCM: takotsubo cardiomyopathy; COPD: chronic obstructive pulmonary disease; DM2: type 2 diabetes mellitus; HTN: hypertension; HIV: human immunodeficiency virus

Variable	Patients without VT		Patients with VT		p-value
COPD	12869	(33.7)	577	(30.0)	0.001
Iron deficiency anemia	1645	(4.3)	76	(3.9)	0.449
Coagulopathy	1098	(2.9)	106	(5.5)	0.001
Cerebrovascular disease	549	(1.4)	42	(2.2)	0.008
DM2	8652	(22.7)	435	(22.6)	0.956
HTN	13998	(36.6)	512	(26.6)	0.001
Alcohol use disorder	2176	(5.7)	156	(8.1)	0.001
Liver disease	893	(2.3)	77	(4.0)	0.001
Peripheral vascular disease	893	(2.3)	62	(3.2)	0.013
Atrial fibrillation	7703	(20.2)	566	(29.4)	0.001
Hypothyroidism	6828	(17.9)	266	(13.8)	0.001
HIV	87	(0.2)	2	(0.1)	0.260
Coronary artery disease	14697	(38.5)	732	(38.0)	0.691
Pulmonary HTN	2540	(6.6)	173	(9.0)	0.001
Tobacco use disorder	388	(1.0)	14	(0.7)	0.215
Obstructive sleep apnea	2156	(5.6)	132	(6.9)	0.025
Opioid use disorder	1205	(3.2)	58	(3.0)	0.728
Obesity	4382	(11.5)	235	(12.2)	0.324
End-stage renal disease	762	(2.0)	56	(2.9)	0.006

A summary of the crude analysis of outcomes of TCM patients based on VT status is included in Table 3. Patients with VT had a higher mortality rate during hospitalization compared to patients without VT (272 (14.1%) vs. 2227 (5.8%); p=0.001). TCM patients who developed VT also had a higher prevalence of complications including cardiac arrest (312 (16.2%) vs. 961 (2.5%); p=0.001), atrioventricular block (211 (11%) vs. 1885 (4.9%); p=0.001), mechanical ventilation (152 (7.9%) vs. 2100 (5.5%); p=0.001), vasopressor use (106 (5.5%) vs. 823 (2.2%); p=0.001), and ST-elevation myocardial infarction (STEMI) (78 (4.1%) vs. 1203 (3.1%); p=0.028). VT patients underwent more procedures including left heart catheterization (851 (44.2%) vs. 15619 (40.9%); p=0.004), right heart catheterization (RHC) (26 (1.4%) vs. 274 (0.7%); p=0.002), intra-aortic balloon pump (IABP) insertion (98 (5.1%) vs. 467 (1.2%); p=0.001), and angioplasty (86 (4.5%) vs. 939 (2.5%); p=0.001). Independent samples T-test analysis showed that the length of hospital stay was significantly longer in those with VT compared to those without (10.23 days vs. 6.67 days; p=0.001), and the total charges of hospitalization were higher ($174615 vs. $96539; p=0.001).

**Table 3:** Outcomes for the study population of TCM patients with and without VT The data has been represented as n (%). p-values are significant at <0.05. VT: ventricular tachycardia; TCM: takotsubo cardiomyopathy; AV: atrioventricular; STEMI: ST-elevation myocardial infarction; NSTEMI: non-ST-elevation myocardial infarction

Variable	Patients without VT		Patients with VT		p-value
Died during hospitalization	2227	(5.8)	272	(14.1)	0.001
Length of stay (days)	6.67		10.23		0.001
Total charges ($)	96539		174615		0.001
Cardiac arrest	961	(2.5)	312	(16.2)	0.001
Permanent pacemaker placement	3914	(10.2)	221	(11.5)	0.082
Cardiac tamponade	68	(0.2)	7	(0.4)	0.066
Acute pericarditis	124	(0.3)	8	(0.4)	0.497
AV block	1885	(4.9)	211	(11.0)	0.001
Angioplasty	939	(2.5)	86	(4.5)	0.001
Intracardiac thrombus	286	(0.7)	21	(1.1)	0.093
Pulmonary embolism	739	(1.9)	49	(2.5)	0.060
Right heart catheterization	274	(0.7)	26	(1.4)	0.002
Left heart catheterization	15619	(40.9)	851	(44.2)	0.004
Intra-aortic balloon pump	467	(1.2)	98	(5.1)	0.001
Mechanical ventilation	2100	(5.5)	152	(7.9)	0.001
Vasopressor use	823	(2.2)	106	(5.5)	0.001
STEMI	1203	(3.1)	78	(4.1)	0.028
NSTEMI	8998	(23.6)	437	(22.7)	0.387

**Figure 1:**
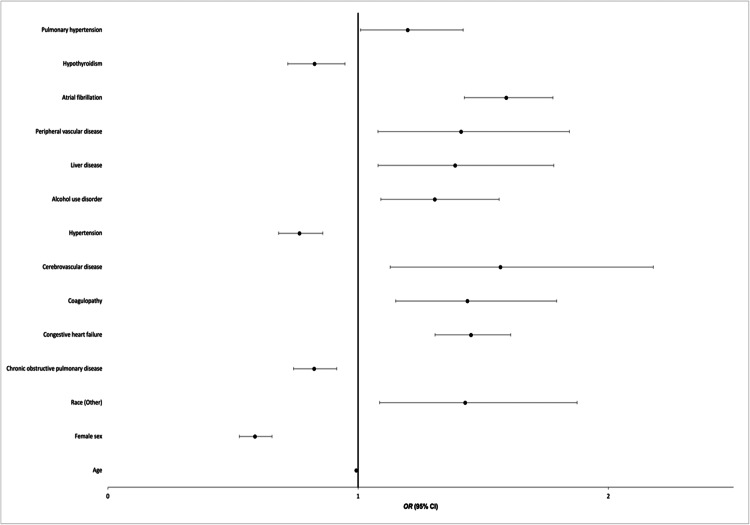
Multivariate logistic regression to assess predictors of VT among patients with TCM VT: ventricular tachycardia; TCM: takotsubo cardiomyopathy; OR: odds ratio; CI: confidence interval

We conducted a multivariate logistic regression to evaluate the predictors of VT among TCM patients as summarized in Figure 1. A demographic predictor that was significantly associated with an increased risk of VT in TCM patients was patients who identified as Other (adjusted OR (aOR): 1.427; 95% CI: 1.086-1.875; p=0.011). Female sex (aOR: 0.587; 95% CI: 0.526-0.656; p=0.001) and age (aOR: 0.992; 95% CI: 0.989-0.996; p=0.001) were protective against VT among TCM patients. Comorbidities and outcomes significantly associated with an increased risk of VT in TCM patients were atrial fibrillation (AF) (aOR: 1.592; 95% CI: 0.00-1.424; p=0.001), congestive heart failure (CHF) (aOR: 1.451; 95% CI: 1.307-1.610; p=0.001), coagulopathy (aOR: 1.436; 95% CI: 1.150-1.793; p=0.001), alcohol use disorder (aOR: 1.306; 95% CI: 1.091-1.563; p=0.004), cerebrovascular disease (aOR: 1.568; 95% CI: 1.128-2.180; p=0.007), peripheral vascular disease (aOR: 1.411; 95% CI: 1.079-1.844; p=0.012), liver disease (aOR: 1.387; 95% CI: 1.080-1.782; p=0.010), and pulmonary hypertension (aOR: 1.197; 95% CI: 1.010-1.420; p=0.03). Conditions that were protective against VT included COPD (aOR: 0.824; 95% CI: 0.742-0.915; p=0.001), hypertension (aOR: 0.765; 95% CI: 0.682-0.858; p=0.001), and hypothyroidism (aOR: 0.825; 95% CI: 0.719-0.947; p=0.006).

**Figure 2:**
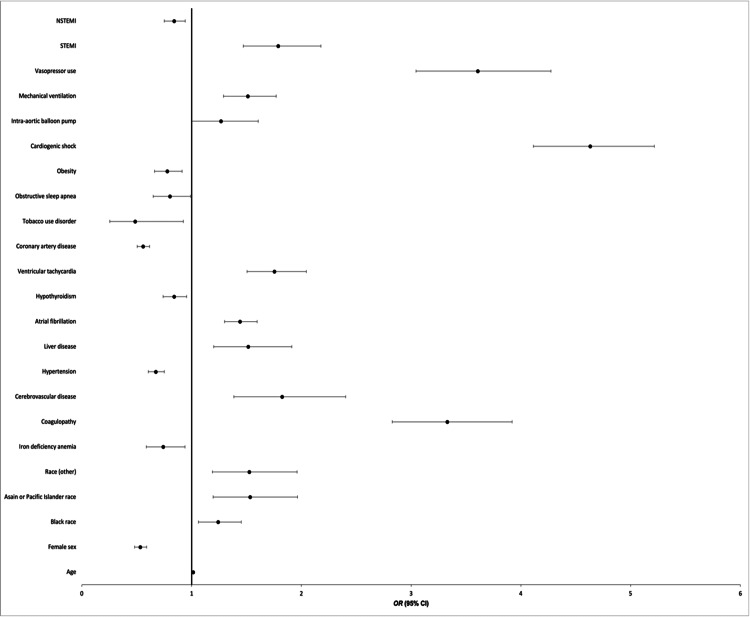
Predictors of mortality in TCM patients TCM: takotsubo cardiomyopathy; OR: odds ratio; CI: confidence interval

A multivariable logistic analysis was constructed to evaluate the predictors of mortality among patients admitted with takotsubo cardiomyopathy as illustrated in Figure 2. Age was found to be one of the factors that increased the odds of mortality in TCM (aOR: 1.014; 95% CI: 1.011-1.018; p=0.001). On the basis of sex, females were associated with a decreased likelihood of mortality (aOR: 0.532; 95% CI: 0.480-0.590; p=0.001). Among indicators of race, mortality was significant among patients who identified as Asian or Pacific Islander (aOR: 1.533; 95% CI: 1.197-1.964; p=0.001), Black (aOR: 1.242; 95% CI: 1.062-1.452; p=0.007), and Other (aOR: 1.526; 95% CI: 1.188-1.961; p=0.001). When stratified according to comorbidities and outcomes, we found predictors of mortality to include coagulopathy (aOR: 3.339; 95% CI: 2.827-3.921; p=0.001), cerebrovascular disease (aOR: 1.824; 95% CI: 1.385-2.404; p=0.001), liver disease (aOR: 1.516; 95% CI: 1.201-1.913; p=0.001), AF (aOR: 1.441; 95% CI: 1.301-1.597; p=0.001), STEMI (aOR: 1.789; 95% CI: 1.470-2.177; p<0.001), VT (aOR: 1.754; 95% CI: 1.505-2.045; p=0.001), cardiogenic shock (aOR: 4.633; 95% CI: 4.114-5.217; p=0.001), intra-aortic balloon pump (IABP) (aOR: 1.268; 95% CI: 1.001-1.607; p=0.050), vasopressor use (aOR: 3.607; 95% CI: 3.045-4.272; p=0.001), and mechanical ventilation (aOR: 1.512; 95% CI: 1.291-1.771; p=0.001). Some comorbidities that were protective against mortality in TCM included hypertension (HTN) (aOR: 0.674; 95% CI: 0.605-0.751; p=0.001), coronary artery disease (CAD) (aOR: 0.558; 95% CI: 0.504-0.617; p=0.001), tobacco use disorder (aOR: 0.558; 95% CI: 0.255-0.925; p=0.028), obesity (aOR: 0.778; 95% CI: 0.663-0.913; p=0.002), iron deficiency anemia (aOR: 0.742; 95% CI: 0.586-0.939; p=0.013), obstructive sleep apnea (aOR: 0.803; 95% CI: 0.651-0.990; p=0.028), hypothyroidism (aOR: 0.841; 95% CI: 0.740-0.956; p=0.008), and non-STEMI (NSTEMI) (aOR: 0.841; 95% CI: 0.751-0.942; p=0.003). 

## Discussion

We found the prevalence of developing VT among patients admitted with TCM to be 4.8%. Predictors of developing VT among this population included conditions such as AF and CHF. We found the female sex to be not only protective against VT but also protective against mortality among patients admitted with TCM.

In the analysis of patients with TCM, those with VT were found to be more likely to have increased mortality. This is consistent with previous studies on TCM, where patients with cardiac arrhythmias were found to have a higher mortality rate than patients with no arrhythmias [7,8]. Several studies have found that the hospitalization of TCM patients complicated with life-threatening arrhythmia reported worse outcomes [4,9]. Among the cohort of patients who developed VT, 29.4% of them had concomitant AF. After adjusting for confounding variables, the results showed that TCM patients with AF were more likely to have VT. In our study, AF was also found to be one of the predictors of mortality among TCM patients. This is consistent with a large nationwide study of admitted patients with TCM, which found AF to be a risk factor for cardiac arrest and VT [10]. Alongside other studies that have found AF to be associated with worse outcomes, the results suggest that VT and AF can potentially be used as tools for risk stratification in TCM patients [11-14]. Furthermore, studies have demonstrated that TCM may be a risk factor for developing new-onset arrhythmias, with one study finding that a third of their TCM patients developed new-onset AF [11].

Our study also found that patients with TCM had a higher burden of coagulopathy and that coagulopathy was an independent predictor of mortality among TCM patients. This finding is consistent with previous studies, which demonstrated that the prevalence of acute thromboembolic events is higher than expected in TCM and is associated with a high long-term mortality rate [15]. A systematic review conducted by Gregorio et al. has shown that among TCM patients, left ventricular thrombus was found in 2.5% of patients [16]. This is not surprising given that TCM is characterized by low blood flow in the ventricles, which predisposes to thrombus formation. As a result, it has been suggested that anticoagulation be initiated for all TCM patients at high risk for thromboembolism [15].

On the basis of demographics, our data showed that TCM patients who identified as Asian, Pacific Islander, or Black were at higher odds of mortality. This is consistent with the current literature that showed Black patients with TCM had higher rates of in-hospital adverse events and mortality [17]. The same study also found that inpatient mortality was higher for male patients who identified as Black than among females who identified as White [17]. While our study did not conduct an analysis on race and sex combined, on the basis of sex alone, our data found that females with TCM had lower mortality compared to males. In another study that analyzed racial differences in TCM outcomes, Black patients were found to have increased in-hospital complications [18]. Moreover, a study that examined ethnic disparities in TCM using data from the International Takotsubo Registry found that Japanese patients had higher in-hospital mortality and required more interventions, such as vasopressor or IABP use [19] This is consistent with our findings that Asian or Pacific Islander patients experienced higher mortality rates. With the limited data and analyses on the ethnic disparities that exist in TCM patients, further studies are needed to examine the reasons behind higher mortality rates in certain races compared to others.

Limitations of our study include that our data was derived from the National Inpatient Sample database, which is an administrative database prone to coding errors. Secondly, although we accounted for confounders in our logistic regression, there may exist other confounders such as medication use that the database does not provide. Lastly, patients in our analysis were not followed longitudinally, therefore long-term outcomes cannot be assessed. 

## Conclusions

TCM is a transient form of heart failure induced by physical or emotional stress, which, in rare cases, can be complicated by arrhythmias such as VT. In our study, we found the incidence of VT among TCM patients to be 4.8%. Predictors of VT included conditions such as AF and coagulopathy. The female sex was found to be protective against VT and mortality in TCM patients. In addition, we found Black, Asian, and Pacific Islander race to be predictors of mortality among TCM patients. Future research should aim to more closely examine the relationship that exists between TCM and fatal arrhythmias such as VT. 
